# Recovery of Extracellular Polymeric Substances from Excess Sludge Using High-Flux Electrospun Nanofiber Membranes

**DOI:** 10.3390/membranes13010074

**Published:** 2023-01-07

**Authors:** Da-Qi Cao, Xiao-Dan Liu, Jia-Lin Han, Wen-Yu Zhang, Xiao-Di Hao, Eiji Iritani, Nobuyuki Katagiri

**Affiliations:** 1 Sino-Dutch R&D Centre for Future Wastewater Treatment Technologies/Key Laboratory of Urban Stormwater System and Water Environment, Beijing University of Civil Engineering and Architecture, Beijing 100044, China; 2 Institute of Soil Environment and Pollution Remediation, Beijing Municipal Research Institute of Environmental Protection, Beijing 100037, China; 3 Department of Chemical Engineering, Nagoya University, Furo-cho, Chikusa-ku, Nagoya 464-8603, Japan; 4 Department of Environmental Technology, Meijo University, 1-501 Shiogamaguchi, Tempaku-ku, Nagoya 468-8502, Japan

**Keywords:** extracellular polymeric substance, recovery, electrospun nanofiber membrane, dead-end filtration, heavy-metal ion

## Abstract

The recycling of extracellular polymeric substances (EPSs) from excess sludge in wastewater treatment plants has received increasing attention in recent years. Although membrane separation has great potential for use in EPS concentration and recovery, conventional membranes tend to exhibit low water flux and high energy consumption. Herein, electrospun nanofiber membranes (ENMs) were fabricated using polyvinylidene fluoride (PVDF) and used for the recovery of EPSs extracted from the excess sludge using the cation exchange resin (CER) method. The fabricated ENM containing 14 wt.% PVDF showed excellent properties, with a high average water flux (376.8 L/(m^2^·h)) and an excellent EPS recovery rate (94.1%) in the dead-end filtration of a 1.0 g/L EPS solution at 20 kPa. The ENMs displayed excellent mechanical strength, antifouling properties, and high reusability after five recycles. The filtration pressure had a negligible effect on the average EPS recovery rate and water flux. The novel dead-end filtration with an EPS filter cake on the ENM surface was effective in removing heavy-metal ions, with the removal rates of Pb^2+^, Cu^2+^, and Cr^6+^ being 89.5%, 73.5%, and 74.6%, respectively. These results indicate the potential of nanofiber membranes for use in effective concentration and recycling of EPSs via membrane separation.

## 1. Introduction

Resource recovery from wastewater has received increasing attention for application in sustainable wastewater treatment systems [1,2]. In such systems, the handling of excess sludge accounts for almost half of the total investment and operational costs of wastewater treatment plants (WWTPs) [3,4,5], wherein excess sludge is the main source of resource materials [6,7,8,9,10,11]. If these resource materials can be recycled, the burden of sludge treatment will be reduced, thereby contributing to a circular economy and a circular society [7,10,11,12].

As a high-value-added product of excess sludge, extracellular polymeric substances (EPSs) are derived from the secretion of microbial cells and cell autolysis, and they mainly consist of polysaccharides, proteins, lipids, humus, nucleic acids, and DNA, accounting for 10–40% of the sludge dry weight [8,13]. The recovered eco-friendly EPSs can be used as adsorbents for heavy-metal ions [7,10,14], flame retardants [15,16], soil conditioners [17], and bioflocculants [18,19]. In particular, the EPSs in aerobic granular sludge (AGS), a recent innovation in biofilm reactors for wastewater treatment, are mainly composed of alginate; they have higher added value than the commonly used EPSs recovered from various sludge in conventional wastewater treatments, such as the sequencing batch reactor technology, as well as the anaerobic or anoxic/oxic, and anaerobic/anoxic/oxic processes [1,6,9,11]. In addition, the removal of EPSs from excess sludge is conducive to sludge concentration and dehydration, agricultural use, incineration, and subsequent disposal [8,17,19]. However, the high moisture contents of EPS solutions extracted from sludge constitute the main bottleneck in the recovery process; thus, it is necessary to explore new technologies for EPS concentration [7,10].

Direct drying methods, such as heating and drying, are generally undesirable for this process due to their high energy consumption and potential generation of secondary pollution, not to mention the possibility that the polymer structures can be damaged during such processes [7]. Membrane separation technologies, therefore, represent possible alternatives for the separation and purification of such substances because of their high separation efficiencies and lack of secondary pollution [20,21,22]. In previous studies, the effective separation of polysaccharides and proteins was achieved by ultrafiltration [7,9], and the effective recovery of EPSs without damage or the generation of secondary pollution was possible [10]. However, EPSs are mixtures of various macromolecular substances; therefore, depending on the membrane characteristics, the EPSs can produce greater degrees of membrane fouling via pore clogging, foulant adhesion, foulant layer formation, and consolidation [23]. In addition, traditional filtration membranes possess a number of inherent limitations, such as a low flux and a susceptibility to pollution due to the regular void arrangement on the transverse membrane surface and the vertical pore arrangement [24,25].

To address such issues, the electrospinning approach has been considered for membrane preparation. This process involves the application of a strong electric field to generate nanofibers from a charged polymer solution [26,27,28,29,30]. As a result, electrospun nanofiber membranes (ENMs) with random interconnected pores can be formed from overlapping fibers [31,32,33], which provide interconnected structures with high porosities that allow liquids to pass through multiple channels [34,35]. In addition, ENMs tend to exhibit a superior fouling resistance to traditional membranes, thereby mitigating the blockage of a single membrane pore and resulting in more promising filtration applications [24,36,37]. For example, a polyvinylidene fluoride (PVDF) ENM with pore sizes <200 nm was used to fabricate the bases of thin-film composite membranes, giving a high flux of 993 L/(m^2^·h), which is three times that of the conventional casting membrane [38]. Another PVDF nanofiber membrane fabricated by three-dimensional printing and near-field electrospinning showed an excellent filtration performance with a high flux of 1020.7 L/(m^2^·h) and a particle rejection of 96.7% when an aqueous dispersion of SiO_2_ (average particle size = 50 μm) was employed as the feed solution [32]. Even for natural river water, a high flux of 20,455 L/(m^2^·h) was obtained at a rejection rate of 99.8% for river particles measuring ~2 μm in diameter using electrospun fibrous cellulose acetate membranes [39]. Furthermore, a nanoparticle rejection rate of ~99.0% was achieved in the filtration of nanoparticle suspensions [40]. Overall, ENMs have been shown to exhibit superior mechanical properties and high specific surface areas, in addition to permitting the facile modification of their functional groups to provide unique functionalities [34,41], thereby rendering them excellent filtration materials [42].

The unique nanostructures of ENMs not only facilitate membrane modification but also provide the conditions for the formation of free nanogaps with directional nanochannels to permit substance transport, thereby resulting in the selective separation and concentration of polymeric substances [22,42,43,44]. For example, an ENM fabricated by the hot-pressing of PVDF and polyacrylonitrile presented high rejection percentages for bovine serum albumin and bovine γ-globulin molecules, i.e., 60% and 75%, respectively [43]. In addition, a nanofibrous membrane composed of a hollow porous polystyrene/ethylene–vinyl acetate copolymer blend was generated by electrospinning and chemical modification and was subsequently used to separate and purify proteins with a 94.35% rejection rate of serum albumin [22]. Furthermore, the excellent separation performances of ENMs are of particular interest in the medical field [42]. For example, a dual-layer nanofiber membrane composed of a polydopamine/polyacrylonitrile layer and a chitosan/sericin layer achieved creatinine and urea clearance rates of 82.3 and 92.8%, respectively, while retaining 83.9% of bovine serum albumin [37]. Moreover, a polymeric hemodialysis membrane composed of an electrospun polyacrylonitrile nanofibrous support layer achieved a 98.4% retention of bovine serum albumin after 4 h [45]. In addition, PVDF, which has excellent mechanical and thermal strength, is a semi-crystalline polymer mainly composed of 59.4% fluorinated chains and synthesized by free-radical polymerization [31]. Furthermore, these fluorinated chains on the surface of PVDF impart chemical and aging resistance [30,46]. Due to these characteristics, PVDF has been widely used in the development of novel nanofiber membranes [27,30,31,32,35,38,41,43,44]. Table 1 lists the typical parameters associated with a selection of ENMs fabricated using PVDF over the past 5 years.

Heavy metals are responsible for reducing the quality of water owing to their high toxicities, their ability to bioaccumulate, and their nonbiodegradable nature [10,53,54]. Thus, ENMs have been examined for their application in the removal of heavy metals [55,56]. For example, metal–organic frameworks were enmeshed in PVDF and polyacrylonitrile electrospun nanofibers to produce nanofibrous membranes for the treatment of Pb^2+^ solutions [57], while PVDF nanofibrous membranes hybridized with silicon oxide nanoparticles exhibited a Cu^2+^ adsorption of ~21.9 mg/g [58]. In addition, the incorporation of carbohydrate polymers, such as chitosan, cellulose, alginate, pullulan, starch, and hyaluronic acid into nanofibrous structures via the electrospinning approach has also been proven effective in the adsorption of heavy metals [49,59]. More specifically, a polydopamine-containing PVDF nanofiber membrane achieved a Cu^2+^ adsorption capacity of 26.7 mg/g [60], in addition to an adsorption capacity of 126.7 mg/g for Cr^6+^ [61]. Furthermore, a multilayer electrospun nanofibrous membrane composed of chitosan and polyvinyl alcohol was used for the dynamic removal of Cu^2+^ from synthetic wastewater, giving a rejection rate of up to 98.6% [62].

Herein, the novelty of the present study is to first apply PVDF ENMs with a high water flux, to separate and concentrate EPSs that are extracted from the excess sludge using the cation exchange resin (CER) method. The simplest filtration process, i.e., dead-end filtration (DEF), was carried out to eliminate influence of hydraulic conditions. Initially, the key properties of the PVDF ENMs are analyzed; subsequently, the effects of different PVDF mass fractions on the EPS recovery efficiency and the water flux are investigated and compared with those of typical commercial filtration membranes. In addition, the influence of the applied pressure on the ENM filtration of EPS is also investigated, along with the fouling and reusability of the ENMs. Lastly, the removal of heavy-metal ions (HMIs) via DEF with the EPS filter cake formed on the ENM surface (EPS–ENM–DEF) is confirmed and evaluated, and the advantages of removing HIMs using EPS–ENM–DEF are analyzed.

## 2. Materials and Methods

### 2.1. Materials

Polyvinylidene fluoride (PVDF, HSV900, *M*_W_ ≈ 1000 kDa, Arkema Inc., Paris, France) was used as the electrospinning polymer. *N,N*-Dimethylacetamide (DMAc, analytical reagent, 99.5%) and acetone (analytical reagent, 99%) were used as organic solvents and were purchased from the Rhawn Chemical Reagent Co., Ltd., and the Beijing Reagent Co., Ltd., respectively. PbCl_2_, CuCl_2_⋅H_2_O, and K_2_CrO_7_ (analytical grade) were purchased from Sigma Aldrich (St. Louis, MO, USA). H_2_SO_4_ (98 wt.%), HNO_3_ (98 wt.%), and phenol (C_6_H_5_OH) were obtained from the Beijing Reagent Co., Ltd. Ultrapure water (resistivity ≥18.2 MΩ) was obtained by purifying tap water using an Arium Comfort II ultrapure water system for laboratory use (Sartorius Corp., Göttingen, Germany). The cellulose ultrafiltration membranes (molecular weight cutoffs [MWCOs] = 10 and 100 kDa) were purchased from Millipore Corp. (Billerica, MA, USA). Filter paper (pore diameters = 4 and 7 μm) was purchased from Advantec Corp., Tokyo, Japan. The disposable polyethersulfone filter membrane (0.45 μm) was purchased from the Jinteng Experimental Equipment Co., Ltd., Tianjin, China. The cation exchange resin (CER, Amberlite IR 120 Na) was obtained from Rohm and Haas Corp., Philadelphia, PA, USA. The dialysis bag (MWCO = 3500) was obtained from Viskase Corp., Lombard, IL, USA. Excess sludge was obtained from Beijing Dongba WWTP.

### 2.2. Fabrication of the PVDF Nanofiber Membranes

**Figure 1:**
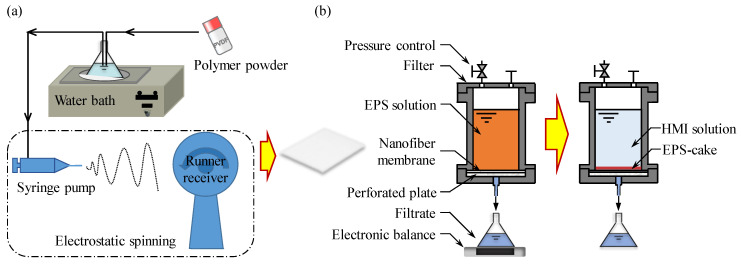
Schematic representations of (**a**) the nanofiber membrane fabrication process, and (**b**) the dead-end filtration apparatus and the experimental process employed herein.

The PVDF nanofiber membranes were prepared via the electrospinning technique (ET2535, Beijing Yongkang Leye Co., Ltd., Beijing, China) with a high-voltage supply of 10–12 kV. The PVDF powder (1.5 g; 14, 18, and 22 wt.%) was initially dissolved in a mixture of DMAc and acetone with a volume ratio of 4:1 (the volumes of the mixture were 8.0, 6.0, and 4.6 mL, respectively) at 300 rpm and 60 °C for 4 h in a constant-temperature water bath (Changzhou Gaode Instrument Manufacturing Co., Ltd., Changzhou, China). Subsequently, each solution was defoamed using a circulating water vacuum pump (SHZ-D(III), GongYi city RuiDe Instrument and Equipment Co., Ltd., Zhengzhou, China) and cooled prior to electrospinning. The desired polymer solution was then transferred into a plastic syringe and delivered at a rate of 4 mL/h using a syringe pump (G20, ID = 0.60 mm). The applied voltage was optimized and fixed at 10.5 kV (+8.0/−2.5 kV) for each membrane fabrication. The drum was covered with aluminum foil at a distance of 20 cm from the syringe needle, and the temperature and humidity conditions inside the electrospinning chamber were set at 22–25 °C and 38–42% relative humidity, respectively. The samples were then cleaned repeatedly with deionized water to remove any residual solvent and give the desired PVDF nanofiber membranes. Finally, the prepared sample was cut into a circular film with a diameter of 6 cm using a custom circular cutter. The fabrication process is illustrated in Figure 1a. The obtained ENMs were denoted as ENM-14, ENM-18, and ENM-22 according to the wt.% of PVDF employed for their preparation. It should be noted that the pre-experiments were conducted under various conditions, such as a PVDF mass ratio of 10–24 wt.% and a spinning rate of 4–10 mL/h; however, only the key results are presented in the manuscript.

### 2.3. Sample Preparation

The EPSs were extracted from the excess sludge sample via CER method [7]. Excess sludges were centrifuged at 4000× *g* and 4 °C for 20 min. Subsequently, the supernatant was removed, and the sediment was collected for experimental analysis and freeze-dried. First, sludge suspensions were prepared by dispersing 1 g of the obtained dry sludge samples in 250 mL of ultrapure water. The corresponding resin (70 g/g volatile solids; VS) was placed into the sludge suspension, and the mixture was stirred at 500 rpm for 4 h. Subsequently, the sludge suspensions were centrifuged at 4000× *g* and 4 °C for 20 min. Each supernatant was dialyzed in a dialysis bag (MWCO = 3500 Da) against a ninefold volume of DI water for 24 h, and the process was repeated two more times to remove all impurities such as various heavy-metal ions that could have been present. The sediment was discarded. Finally, EPS powder was produced by freeze-drying the solution in the dialysis bag. Notably, the EPS powders produced were afforded in quantities that were sufficient for the experiments performed, allowing the prepared batches to be used in all experiments, thus ensuring consistent EPS characteristics.

It was confirmed that this technique allowed recovery of the EPSs because of negligible destruction to the microbial cells in the excess sludge [7,10]. Therefore, these samples can be considered as mixed water-soluble polymers. The EPS was dissolved in ultrapure deionized (DI) water at 24 °C and stirred for 4–12 h to obtain the desired EPS solution. All samples were used immediately to prevent any errors caused by microbial consumption of the organic matter present in the aqueous solution [23,63].

### 2.4. Two-Stage ENM Filtration

The EPS solutions were filtered using a custom-made pressure filter device, as shown in Figure 1b. The two-stage filtration process was carried out as described previously [10]. Herein, the first step involved concentration of the EPSs, wherein the fabricated ENMs were used to filter the EPS solutions (50 mL) to form a dense EPS filter cake layer on the surface of the membrane at a filtration pressure (*p*_1_) of 20 kPa. An ENM filter membrane with an EPS cake layer was obtained as a result of this process. The second step involved the removal of HMIs. More specifically, heavy-metal-containing wastewater samples (10 μM solutions of Pb^2+^, Cu^2+^, and Cr^6+^) were filtered through the cake at a filtration pressure (*p*_2_) of 20 kPa. The HMIs were removed by the synergistic effect of the EPS filter cake and the ENM. For comparison, the ENMs were also used to remove the HMIs without the presence of an EPS filter cake, which can be considered to reflect the adsorption of HIMs by the ENM. Notably, to eliminate the effects of acid, base, or buffers, the pH was not adjusted in any experiments; it was maintained at 6.5 obtained using a pH meter (Mettler Toledo FE20).

### 2.5. Analytical Methods

#### 2.5.1. Membrane Characterization

The morphological properties of the membrane surface and the cross-sectional areas of the PVDF nanofiber membranes were characterized using scanning electron microscopy (SEM, G300, Zeiss, Jena, German) at 2–3 kV. All samples were cut into dimensions of 1 cm × 1 cm and were pretreated with gold spraying for 10 min under vacuum. The electron microscope images were observed at 5–50 k. Image Pro-Plus 6.0 software was used to visualize the SEM images and give the fiber diameters of the ENMs. The porosities and pore sizes of the membranes were obtained by mercury porosity experiments (AutoPore Iv 9510, Micromeritics Instrument Co., Ltd., Norcross, GA, USA), while a thickness gauge (HCC–18, Liuling Instrument Factory, Shanghai, China) was used to measure each membrane thickness. The water contact angles of the PVDF ENMs were measured using a contact angle meter (JC2000D4M, Zhongchen Digital Technology Equipment Co., Ltd., Shanghai, China); finally, the mechanical properties of each sample (shaped into strips of dimensions 2 cm × 1 cm) were tested using an electronic universal testing machine (nonmetallic direction) (Inspekt table blue 5 kN, Hegewald & Peschke, Nossen, Germany) at a distance of 20 cm and a tensile rate of 2 mm/min.

#### 2.5.2. Evaluation of the Membrane Resistance and Filtration Behaviors

All experiments employed DEF with an effective area of 19.6 cm^2^ using a self-made pressure filter device (Figure 1b). A constant pressure was applied using N_2_ gas and was controlled using an automatic pressure-regulating valve. The filtrate liquor was collected in a reservoir and placed on an electronic balance connected to a personal computer to record the mass versus time data. The weights were converted to volumes using a density correlation (i.e., mass/density = volume). The filtration rate was obtained through a numerical differentiation of the volume versus time data. The resistance properties of the membranes were examined by means of ultrapure water filtration experiments [6]. The membrane resistance, *R*_m_ was calculated using Equation (1) as follows:

(1) $$R_{m}=\frac{p}{\mu \cdot J},$$

where *p* represents the applied filtration pressure, *μ* is the viscosity of water, and *J* is the filtration rate during the ultrapure water membrane filtration process.

During cake filtration, the relationship between the reciprocal of the filtration rate (d*θ*/d*v*) and the cumulative filtrate volume collected per unit effective membrane area, *v*, is linear in accordance with the Ruth filtration rate equation [6,7]:

(2) $$\frac{d\theta }{dv}=\frac{2}{K_{v}}v+{\left(\frac{d\theta }{dv}\right)}_{m},$$

where *θ* is the filtration time, and *K*_v_ is the Ruth filtration coefficient, indicating the filterability of the feed. In addition, (d*θ*/d*v*)_m_ represents the membrane flow resistance and is an intercept on the (d*θ*/d*v*) axis according to the Ruth plots of (d*θ*/d*v*) as a function of *v*, which is the value of the reciprocal of the filtration rate at the start of filtration prior to cake formation. In this study, the behaviors of the flux decline were analyzed by evaluation of the curved shapes of the Ruth plots (d*θ*/d*v* vs. *v*) [64,65,66].

#### 2.5.3. Reusability of the Nanofiber Membranes

To evaluate the reusability of each nanofiber membrane, equivalent-concentration EPS solutions were filtered through each PVDF nanofiber membrane under a constant pressure of 20 kPa for a total of five times. Between each filtration, the EPS filter cake was removed by physical scraping. The polluted membrane was then placed in a sealed bag. and ultrasonic cleaning (600 W) was carried out for 10 min at 25 °C. It was observed that the membrane fouling was almost completely removed probably because the recovered EPSs obtained via CER were water-soluble. Prior to each subsequent filtration, pure water was passed through the membrane to check for channel blockage, and it was found that *R*_m_ remained relatively constant.

#### 2.5.4. Size Distribution, Fourier-Transform Infrared Spectroscopy, and Zeta Potential

The typical size distribution of the colloidal EPS solution was measured by dynamic light scattering using a Zetasizer Nano ZS90 size analyzer (Zetasizer Nano ZS90, Malvern Co., Ltd., Malvern, UK). To prepare the samples for analysis by Fourier-transform infrared (FTIR) spectroscopy, the EPS sample was obtained by vacuum freeze-drying (FD-1A-50, Beijing Boyikang Laboratory Instruments Co., Ltd., Beijing, China). The samples were then mixed with KBr (1:100 mass ratio) and dried overnight at 120 °C. Finally, the dried samples were ground into a powder using an agate mortar and pestle, and a tablet press was used to prepare the KBr discs required for functional group analysis by FTIR spectroscopy (Nicolet is5, Thermo Scientific, Waltham, MA, USA). The zeta potentials of the EPSs and the membrane surfaces were measured using a solid surface zeta potential analyzer (SurPASS^TM^ 3 Eco, Anton Paar, Graz, Austria). The surface zeta potential was determined as a function of pH in a 0.001 M KCl electrolyte solution upon varying the solution pH from 5 to 7 by the addition of a HCl or NaOH solution through the automatic titration unit of the instrument [67].

#### 2.5.5. Determination of the EPS Recovery and the HMI Removal Efficiencies

A 5 mL portion of the collected filtrate was used to determine the concentrations of the effluent EPSs and HMIs present in the filtrates obtained by ENM filtration and EPS–ENM filtration, respectively. The concentration of polysaccharides (*C*_p_) in the EPS solution or the collected filtrate obtained using different membranes was determined according to the phenol/sulfuric acid method using UV/Vis spectrophotometry (Cary 5000, Agilent Technologies Co., Ltd., Waldbronn, Germany) (UV method) [8]. As a control, the EPS concentration in the EPS solution or the collected filtrate (*C*_e_) was obtained using the standard freeze-drying weighing method [7]. The HMI concentrations (*C*_i_) in the aqueous solutions were measured using inductively coupled plasma (ICP) spectrometry (ICP 7000 Series, Thermo Scientific, Waltham, MA, USA) after mixing the samples with HNO_3_ (1% *v*/*v*) and filtering through a 0.45 μm membrane to meet the sampling requirements. All experiments were repeated at least twice. Thus, the polysaccharide recovery efficiency (*η*_p_ = 1 − *C*_p_/*C*_p0_), EPS recovery efficiency (*η*_e_ = 1 − *C*_e_/*C*_e0_), and HMI removal efficiency (*η*_i_ = 1 − *C*_i_/*C*_i0_) were obtained. In these equations, *C*_p0_ is the initial polysaccharide concentration in the initial EPS solution, *C*_e0_ is the initial EPS concentration, and *C*_i0_ is the initial concentration of the HMI in the wastewater sample.

The average EPS recovery rates (*η*_av_) during DEF of the EPS solution using the fabricated ENMs and commercial membranes were evaluated using both the UV and the weighing methods, wherein the evaluated data were obtained for the cumulative filtrate volume per unit membrane area, *v* = 2 cm. In Figure S1, the average EPS recovery rates obtained using the UV method are compared with those obtained using the weighing method. As shown in the figure, all data fell within the range bounded by the error bars of ±10% depicted by the dotted lines, indicating that the UV method could accurately evaluate the average EPS recovery rate in the DEF of the EPS solution. Hence, the values of *η*_av_ were evaluated using the UV method for the purpose of this study.

## 3. Results and Discussion

### 3.1. Characterization of the Nanofiber Membranes

**Figure 2:**
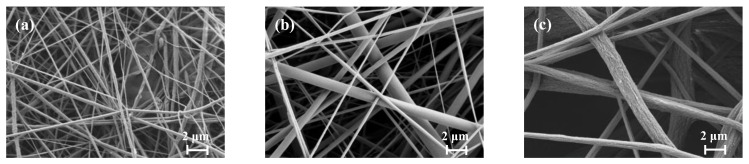
Typical SEM images of the nanofiber membranes obtained using PVDF mass fractions of (**a**) 14 wt.%, (**b**) 18 wt.%, and (**c**) 22 wt.%, denoted as ENM-14, ENM-18, and ENM-22, respectively.

Table 1 also lists the typical morphological and physical parameters of the PVDF ENMs fabricated in this study. In addition, Figure 2 shows the surface morphologies of the ENMs, indicating that a larger PVDF mass fraction resulted in a larger fiber diameter and membrane pore size. More specifically, the fiber diameters and pore diameters of the ENMs were obtained by combining SEM with Image Pro 6.0 image processing software, wherein, as shown in Table 1, the porosity (52.46–73.24%) and the fiber diameter (0.504−1.772 μm) were confirmed to increase with an increase in the PVDF mass fraction [68]. Since the hydrophobic properties of a membrane are known to affect the filtration resistance [69], the water contact angles (WCAs) of the ENMs were measured and are listed in Table 1 (see also Figure S2). As indicated, the WCA increased from 109.5° to 129.3° upon increasing the PVDF mass fraction, and the average WCA was 119.3°; this value corresponds to a typical hydrophobic membrane and results from the high concentration of PVDF fluorine atoms on the ENM surface [70]. Notably, except for the properties of membrane material, the membrane surface roughness also affected the WCA; herein, the porosity of membranes increased with increasing the PVDF mass fraction, resulting in numerous air pockets in the membrane surfaces, leading to higher hydrophobicity (larger WCA) [41].The tensile stress–strain curves of the fabricated ENMs are shown in Figure S3 and Table 1, wherein it can be seen that the tensile strength and nominal tensile strain at break of the ENMs increased upon increasing the PVDF mass fraction because of large fiber diameter and membrane thickness. Of the various samples prepared, ENM-22 exhibited the best mechanical properties due to the higher loading of PVDF, which increased the tensile stress that could be supported by the membrane.

### 3.2. Comparison between the EPS Recovery Properties of the Fabricated Nanofiber Membranes and Commercial Membranes

**Figure 3:**
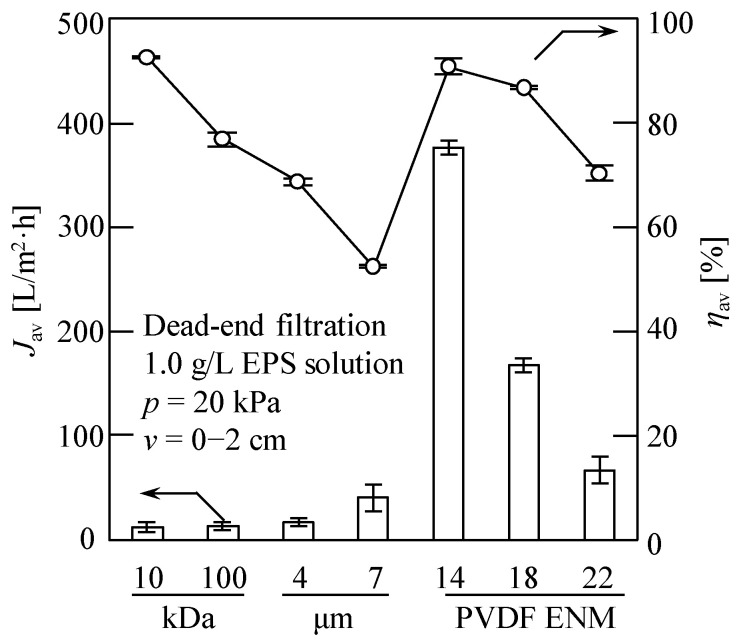
Average water flux (*J*_av_) and average EPS recovery rate (*η*_av_) in the dead-end filtration of a 1.0 g/L EPS solution. A filtration pressure of 20 kPa was used, and the evaluated data were obtained for the cumulative filtrate volume per unit membrane area, *v* = 2 cm. Millipore ultrafiltration membranes (MWCO = 10 and 100 kDa), Advantec filter paper (4 and 7 μm), and the prepared PVDF ENMs (PVDF mass fractions of 14, 18, and 22 wt.%) were evaluated.

The average water flux (*J*_av_) and average EPS recovery rate (*η*_av_) obtained during the DEF of the EPS solution using the fabricated ENMs and commercial membranes are shown in Figure 3, wherein the evaluated data were obtained for the cumulative filtrate volume per unit membrane area, *v* = 2 cm. In general, the nanofiber membranes are ultrafiltration or microfiltration membranes; therefore, herein, the most widely used ultrafiltration membranes (MWCO = 10 and 100 kDa, hydrophobic) and microfiltration membranes (4 and 7 μm, hydrophilic) from Millipore Corp. and Advantec Corp., respectively, were compared. In the case of ENM-14, the highest value of *J*_av_ was achieved (i.e., 376.8 L/(m^2^·h)) in addition to a high *η*_av_ value of 94.1%, while, for the 10 kDa commercial membrane that was previously reported to exhibit a high EPS recovery efficiency (>90%) [7], a significantly lower *J*_av_ of 10.7 L/(m^2^·h) was recorded, although the *η*_av_ value was slightly higher (i.e., 96.4%). These results clearly indicate that the water flux exhibited by ENM-14 was ~35.2 times higher than that of the 10 kDa commercial membrane. It is worth noting that our ENMs were prepared using low-cost PVDF materials, unlike in the cases of commercial ultrafiltration and microfiltration membranes. Therefore, in addition to their high porosities and highly interconnected pore structures that offer a low hydraulic resistance for water transportation and yield a high throughput with a low energy consumption, the ENMs reported herein also impart economic benefits during the recovery and concentration of EPSs and other substances.

**Figure 4:**
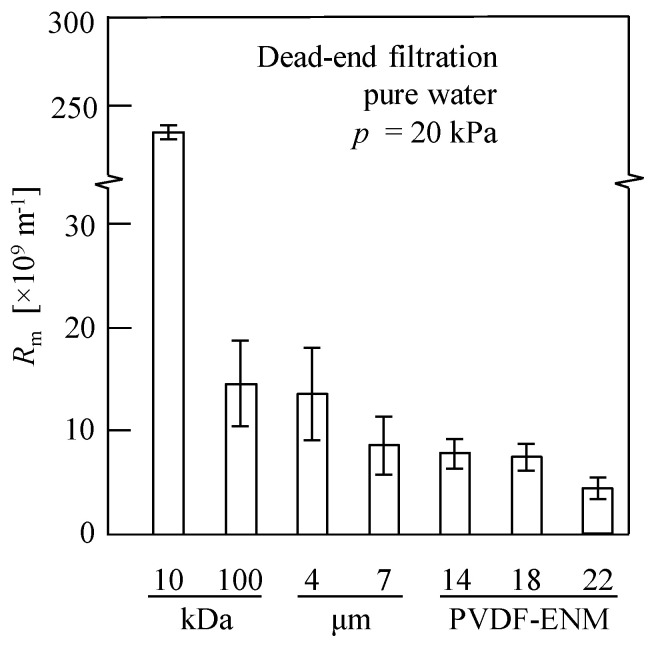
Membrane resistance (*R*_m_) obtained via the dead-end filtration of ultrapure water at a filtration pressure (*p*) of 20 kPa. Millipore ultrafiltration membranes (MWCO = 10 and 100 kDa), Advantec filter paper (4 and 7 μm), and the prepared PVDF ENMs (PVDF mass fractions of 14, 18, and 22 wt.%) were evaluated.

**Figure 5:**
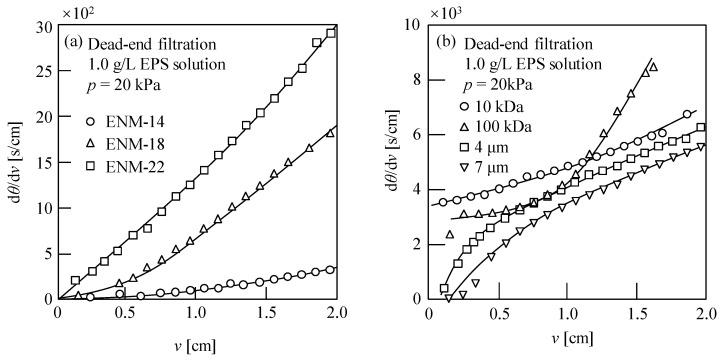
Filtration behaviors during the dead-end filtration of a 1.0 g/L EPS solution at a filtration pressure (*p*) of 20 kPa for (**a**) the ENMs, and (**b**) the commercial membranes. The horizontal axis (*v*) represents the cumulative filtrate volume per unit membrane area, the vertical axis (d*θ*/d*v*) represents the reciprocal of the filtration rate, and *θ* is the filtration time.

Figure 4 shows the resistance values of the ENMs and the commercial membranes. As indicated, for the four commercial membranes, *R*_m_ decreased with increasing membrane pore size, which accounted for the increasing and decreasing *J*_av_ and *η*_av_ values, respectively, as shown in Figure 3. In contrast, for the nanofiber membranes, increases in both *J*_av_ and *η*_av_ values were observed upon decreasing the membrane resistance (see Figure 3 and Figure 4). More specifically, the ENM-14 membrane exhibited both the highest water flux and the highest EPS recovery rate of the various samples investigated, and this may be due to the quasi-three-dimensional network structure of the cross-configuration [68]. Since the EPSs are considered a mixture of various polymers extracted from the excess sludge, large numbers of polysaccharides, proteins, DNA, and other substances are present [7,10]. As a result, the EPSs possess a wide particle size distribution of 6.2–226.7 μm, as shown in Figure S4. Thus, the smaller colloids easily enter the ENM pores, resulting in membrane blockage [65,66]. However, the pore size of ENM-14 with low porosity and small thickness appeared to be sufficiently small to retain the majority of these colloidal particles in the EPSs; in contrast, pore blocking occurred in the ENM-18 and ENM-22 membranes because both Ruth plots (d*θ*/d*v* vs. *v*) exhibited more remarkable downward convex curves (see Figure 5a) based on the membrane pore clogging model [65,66], thereby lowering their EPS recovery rates and water fluxes.

Figure 5 shows the corresponding filtration behaviors of the ENMs and the commercial membranes, wherein it can be seen that the filtration rates of ENMs declined more slowly than those of the commercial membranes. More specifically, the obtained Ruth filtration plots [64], i.e., the relationship between the reciprocal of the filtration rate (d*θ*/d*v*) and the cumulative filtrate volume collected per unit effective membrane area (*v*), show different shapes, i.e., downward convex curves for the ENMs (ENM-14, ENM-18, and ENM-22) and the commercial ultrafiltration membranes (10 and 100 kDa), and upward convex curves for the commercial microfiltration membranes (4 and 7 μm), indicating that the pore blocking mechanisms of nanofiber membranes differ from those of conventional commercial membranes.

### 3.3. Influence of the Applied Pressure on the ENM Filtration of an EPS Solution

**Figure 6:**
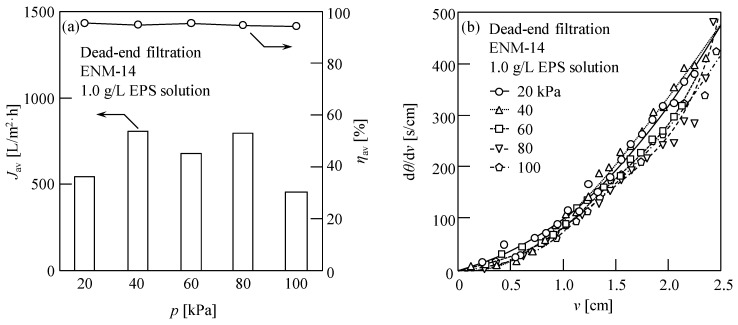
Using the ENM-14 specimen: (**a**) Average water fluxes (*J*_av_), average EPS recovery rates (*η*_av_), and (**b**) filtration behaviors in the dead-end filtration of a 1.0 g/L EPS solution at various filtration pressures (*p* = 20, 40, 60, 80, and 100 kPa). The evaluated data were obtained for the cumulative filtrate volume per unit membrane area, *v* = 2 cm. The horizontal axis shows *v*, while the vertical axis shows the reciprocal of the filtration rate (d*θ*/d*v*), and *θ* is the filtration time.

In general, the filtration rate of a membrane can be enhanced by increasing the applied filtration pressure [7]; therefore, we moved on to examine the influence of the filtration pressure on the concentration and recovery of EPSs using the fabricated ENMs. Thus, Figure 6a shows the influence of *p* on *J*_av_ and *η*_av_ when the EPS solution was subjected to filtration through the ENM-14 specimen. As shown, the average water flux fluctuated upon increasing the pressure from 20 to 80 kPa in steps of 20 kPa. Overall, an increased water flux was observed; however, upon increasing the pressure further to 100 kPa, an overall decrease was observed. These observations were attributed to the high compressibility of the EPS filter cake [7] and blockage of the ENM pores at higher pressures [66]. However, it was also found that the average EPS recovery rate was not affected by the filtration pressure and remained at ~94%. In addition, Figure 6b shows the filtration behavior of a 1.0 g/L EPS solution at various filtration pressures (20–100 kPa), wherein the results confirm the lack of a proportional relationship between the filtration pressure and the water flux, and the Ruth filtration plots show downward convex curves regardless of the filtration pressure employed, which further reveals the membrane pore blocking based on the blocking filtration model [66]. Therefore, to reduce energy consumption, a low filtration pressure was proposed to concentrate and recycle EPSs using these fibrous membranes.

### 3.4. Fouling and Reusability of the ENMs in the Filtration of EPSs

**Figure 7:**
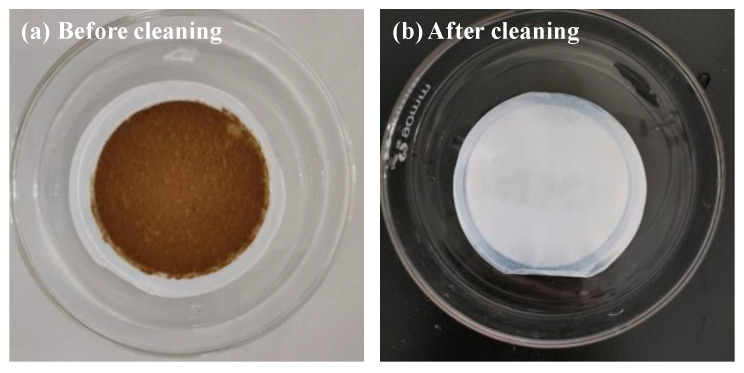
Typical photographic images of the ENMs with recycled and concentrated EPS (**a**) before and (**b**) after ultrasonic cleaning.

**Figure 8:**
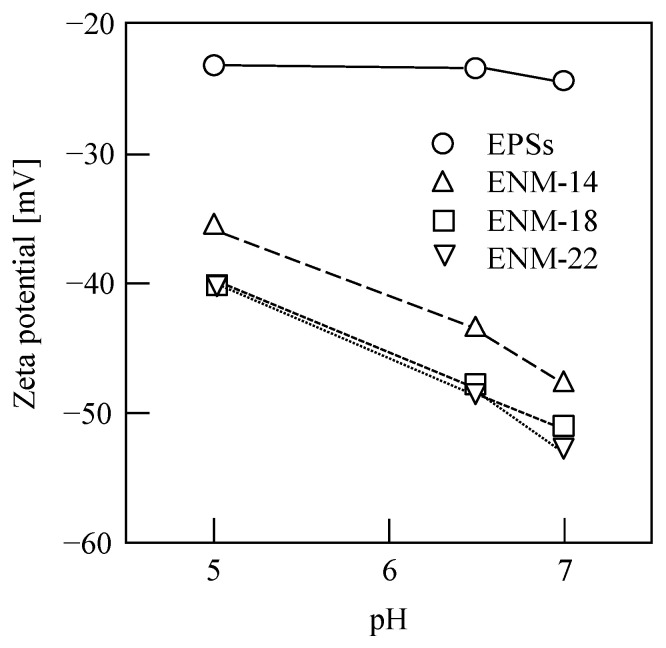
Potentials of the EPS and the ENM membrane surfaces at pH values of 5, 6.5, and 7.

The prevention of membrane fouling is a key factor in the development of new membrane technologies. It is worth emphasizing that the EPSs used in this study were extracted from the excess sludge sample via CER, which is able to efficiently recover the EPSs because of little destruction of microbial cells in the excess sludge [7,10]. Therefore, the EPSs can be considered as mixed water-soluble polymers, and it was found that an EPS filter cake was formed on the membrane surface after filtration. The EPS filter cake was removed by physical scraping before the ultrasonic cleaning of the polluted membrane. Typical images of the ENMs before and after removal of the EPSs by ultrasonic cleaning (600 W, 10 min) are shown in Figure 7, and the polluted membrane with EPS filter cake can be clearly observed (see Figure 7a), indicating that the EPS fouling within the membrane pores and on the membrane surface can be removed using this simple technique because of their water solubility. Meanwhile, Figure 8 shows the zeta potentials of the EPSs and the membrane surfaces of the ENMs at pH values of 5, 6.5, and 7. As shown in the figure, both species exhibit a negative charge regardless of the pH, and the value of zeta potential increases with increasing pH, revealing that electrostatic repulsion between the EPSs and the membranes represents an alternative EPS filtration mechanism. Thus, the EPS cake layer formed in our study was completely removed via simple physical cleaning owing to the water solubility of EPSs and the electrostatic repulsion between the EPSs and the membranes.

**Figure 9:**
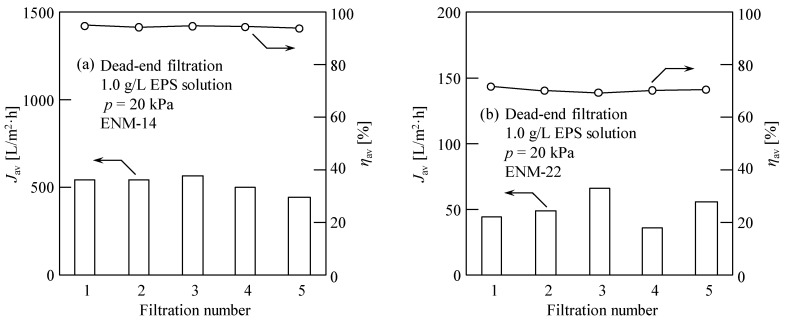
Average water flux (*J*_av_) and average EPS recovery rate (*η*_av_) during the dead-end filtration of a 1.0 g/L EPS solution with a filtration pressure (*p*) of 20 kPa using the (**a**) ENM-14 and (**b**) ENM-22 specimens. Five filtration cycles were carried out wherein the membrane was cleaned using ultrasonic irradiation for 10 min between filtrations. The evaluated data were obtained for the cumulative filtrate volume per unit membrane area, *v* = 2 cm.

Using the ultrasound-based membrane cleaning technique (i.e., ultrasonication for 10 min after each filtration), the filtration experiments were repeated five times using the recycled membrane to evaluate the reusability of the fabricated ENM. In this case, the ENM-14 and ENM-22 membranes were used because they exhibited the lowest and highest degrees of membrane fouling (i.e., the maximum and minimum water fluxes), respectively. Figure 9 shows the average water flux and average EPS recovery rate during the DEF of a 1.0 g/L EPS solution with a filtration pressure of 20 kPa using the ENM-14 and ENM-22 specimens. From this figure, it can be seen that, over five filtration cycles, the average EPS recovery remained relatively constant, as did the average water flux, although there was a slight increase in the latter. These results, therefore, indicate that the fabricated ENMs exhibit a good reusability for the recovery of EPSs extracted from excess sludge via CER.

### 3.5. Removal of HIMs via EPS–ENM Dead-End Filtration

**Figure 10:**
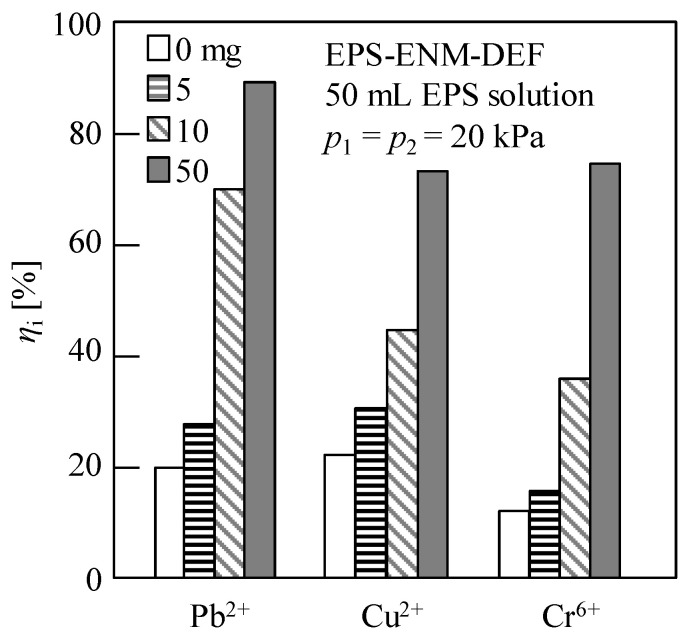
Removal efficiency (*η*_i_) of the heavy-metal ions (HMIs) via dead-end filtration using the ENM-14 with an EPS filter cake on the surface (EPS–ENM–DEF). The evaluated data were obtained for the cumulative filtrate volume per unit membrane area, *v* = 9 cm. First stage: concentration and recovery filtration of 0, 0.1, 0.2, and 1.0 g/L EPS solutions (50 mL) at *p*_1_ = 20 kPa to give EPS filter cakes with masses of 0, 5, 10, and 50 mg, respectively. Second stage: filtration of 10 μM solutions (180 mL) of the desired HIM (Pb^2+^, Cu^2+^, or Cr^6+^, pH 6.2–6.7) at *p*_2_ = 20 kPa.

Subsequently, we carried out a two-stage DEF procedure (see Figure 1b) using the prepared ENM-14 specimen, wherein the first stage involved the above-described recycling and concentration of an EPS solution, and the second stage involved the removal of HIMs from wastewater, i.e., the removal stage of HMIs via EPS–ENM–DEF. The detailed working mechanism for the removal of HIMs via EPS–ENM–DEF can be seen in our previous study [10]. Thus, the removal efficiency achieved using a 10 μM aqueous solution of the desired HIM (Pb^2+^, Cu^2+^, or Cr^6+^, pH 6.2–6.7) by DEF in the presence of the EPS filter cake on the ENM surface (i.e., EPS–ENM–DEF) is shown in Figure 10. It should be noted that the EPS filter cakes were formed in the first stage from 50 mL aliquots of 0, 0.1, 0.2, and 1.0 g/L EPS solutions to give filter cake masses of 0, 5, 10, and 50 mg, respectively. As shown in the figure, the HMI removal efficiencies increased upon increasing the EPS filter cake mass, with removal efficiencies of 89.5%, 73.5%, and 74.6% being achieved for Pb^2+^, Cu^2+^, and Cr^6+^, respectively, using the 50 mg EPS filter cake. Furthermore, adsorption experiments revealed membrane adsorption capacities of 20.6%, 21.8%, and 12.0% for Pb^2+^, Cu^2+^, and Cr^6+^, respectively, thereby indicating that EPSs play a major role in the removal of HIMs, corroborating the results of previous studies [7,10,71,72,73].

**Figure 11:**
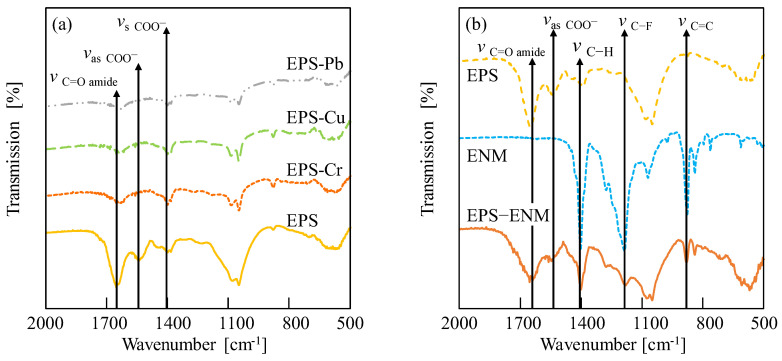
FTIR spectra for the various samples: (**a**) the EPS filter cake before and after the adsorption of HIMs to give EPS-Pb, EPS-Cu, and EPS-Cr; (**b**) the EPS, ENM, and EPS–ENM samples.

Figure 11a shows the FTIR spectra of the EPS filter cake before and after the adsorption of the various HIMs (i.e., EPS-Pb, EPS-Cu, and EPS-Cr). In addition, the spectra of the ENM and the EPS-contaminated ENM (EPS–ENM) are shown in Figure 11b. As indicated in Figure 11a, all the samples appeared to contain similar functional groups corresponding to polysaccharides, proteins, lipids, and nucleic acids, thereby indicating that the HIMs do not alter the molecular structure of EPSs upon their adsorption, and that the interaction mechanisms with the EPSs were similar for all three HIMs. As previously reported for similar experiments carried out using Pb^2+^ [10], the carboxylate residues of the EPSs also appeared to be bridged by the Cu^2+^ and Cr^6+^ ions.

Figure 11b shows the FTIR spectra of the EPS, ENM, and EPS–ENM samples. More specifically, in the case of the EPS–ENM sample, three strong peaks were observed at 1402, 1167, and 882 cm^−1^, which correspond to the C–H, C–F, and C–C moieties of PVDF ENM, respectively [69,74]. Meanwhile, two strong peaks were found at 1658 and 1539 cm^−1^, which correspond to the C=O and COO^−^ groups of EPS, respectively. These results indicate that the EPS and ENM species are tightly bound to one another through electrostatic interactions attributed to the fluorine atoms of the PVDF ENM [75]. The electronegative nature of fluorine also accounts for the successful adsorption of small amounts of HIMs on the ENM specimen in the absence of the EPS filter cake. It is, therefore, expected that numerous mechanisms could be involved in the adsorption of HIMs on these EPS–ENMs, such as electrostatic attractions, complexation, ion exchange, and surface precipitation, thereby indicating the potential for preparing novel fibrous membranes with high HIM adsorption capacities in the future.

**Figure 12:**
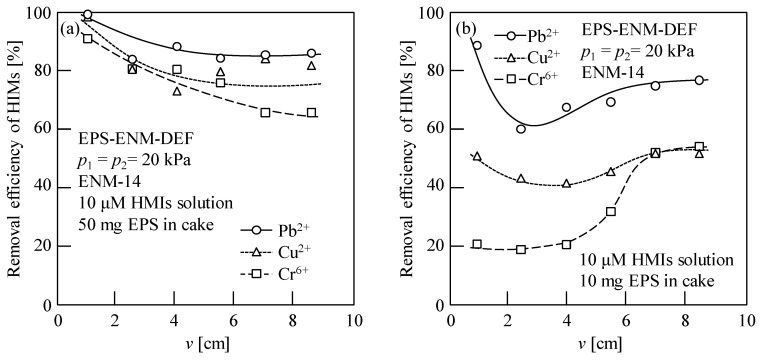
HIM removal efficiencies during the EPS–ENM–DEF process on ENM-14 for (**a**) 50 mg and (**b**) 10 mg EPS filter cakes. First stage: concentration and recovery filtration of 1.0 and 0.2 g/L EPS solutions (50 mL) at *p*_1_ = 20 kPa to give EPS filter cakes with masses of 50 and 10 mg, respectively. Second stage: filtration of 10 μM solutions (180 mL) of the desired HIM (Pb^2+^, Cu^2+^, or Cr^6+^, pH 6.2–6.7) at *p*_2_ = 20 kPa. *v* is the cumulative filtrate volume per unit membrane area.

Furthermore, Figure 12 shows that the HIM removal efficiency varies during the EPS–ENM DEF process, wherein different results were obtained for the 10 and 50 mg EPS filter cakes. More specifically, as shown in Figure 12a, in the presence of a 50 mg EPS filter cake, the removal efficiencies of the three HIMs decreased with the filtration progress, wherein the greatest decline was observed for Cr^6+^, as previously reported [10]. This can be accounted for by considering that, in the initial stage of EPS–ENM–DEF, the EPS filter cake contained numerous vacant adsorption sites; however, during the filtration process, these sites became occupied. In contrast, for the 10 mg EPS filter cake, after an initial decrease, the removal efficiencies of the three HIMs increased prior to becoming stable, as shown in Figure 12b. This was particularly apparent in the case of Cr^6+^, wherein the initially low removal efficiency increased gradually but significantly, and this was attributed to the structural rearrangement of the EPS filter cake upon interaction with Cr^6+^ because of highly charged ions. Figure S5 also shows the HIM removal efficiencies during the EPS–ENM–DEF process on ENM-14 for the 5 and 0 mg EPS filter cakes. It should also be noted that the HIM removal efficiency is also likely influenced by a range of other factors, such as ion rejection by the cake or the ENM, ion adsorption by the ENM, and the filtration rate.

### 3.6. Advantages of Removing HIMs by EPS–ENM Filtration

As described above, the HMI pollution of natural water bodies and terrestrial ecosystems can be attributed to the persistence and bioaccumulation of such elements. Due to these factors, in addition to their toxic nature, HMIs post a great threat to vegetation, crops, and aquatic species [76]. The proposed two-stage EPS–ENM–DEF filtration process for HIM removal, therefore, appears to offer a number of advantages over traditional adsorption materials and processes. More specifically, the EPSs are derived from the excess sludge of wastewater treatment plants, and they have been proven to exhibit excellent adsorption properties [7,10,71,72,73], while also efficiently removing low concentrations of metal ions [77,78]; these factors greatly increase the value of such waste. Furthermore, in terms of complex pollutants, the adsorption capacities of EPSs toward certain multicomponent heavy-metal compositions have been found to be higher than those recorded for their individual single components [78,79], thereby confirming that EPSs appear suitable for dealing with complex water bodies.

Overall, our results indicate that these PVDF nanofiber membranes exhibit a high water flux that is retained during their repeated recycling, thereby permitting them to concentrate EPSs, with a recovery rate of up to 94.1% being achieved over five cycles. This was attributed to the high porosity and highly interconnected porous structure of the prepared ENMs. Table 1 shows a comparison of the typical parameters of various ENMs in the literature fabricated using PVDF, which have been reported over the past 5 years. These ENMs were optimized according to the types or proportions of solvents and modification materials (e.g., nanoparticles, carbon nanotubes, or graphene oxide) [27,31,49,50,52,80], thereby indicating that, in the future, it should be possible to develop superior ENMs that exhibit high water fluxes, strong mechanical properties, high reusability, and good antifouling properties through unique design and processing techniques. In particular, as indicated by the current study, ENMs exhibiting an electronegative surface can attract metal cations via electrostatic interactions, thereby improving their adsorption capabilities [81]. In our system, we found that both the EPS adsorbent and the HIMs were fixed on the ENM, which was easily separable from the clarified effluent, thereby significantly simplifying the post-treatment separation phase [56]. As a result, the application of ENMs to EPS recovery simplifies the practical application of EPSs to remove HIMs from wastewater systems. We, therefore, expect that EPS–ENMs will become an environmentally friendly class of materials for heavy-metal removal.

## 4. Conclusions

We herein reported the development of PVDF electrospun nanofiber membranes (ENMs) exhibiting a high water flux for the separation and concentration of extracellular polymeric substances (EPSs) extracted from excess sludge using the cation exchange resin (CER) method. The ENM prepared using a 14 wt.% loading of PVDF exhibited excellent properties, including a high water flux (376.8 L/(m^2^·h)), which was 35.2 times greater than that achieved using a commercial 10 kDa membrane. In addition, an excellent EPS recovery rate of up to 94.1% was achieved using this ENM for the dead-end filtration (DEF) of a 1.0 g/L EPS solution at 20 kPa. Overall, the prepared ENMs displayed excellent mechanical strengths, antifouling properties, and high reusability, wherein their original performances were almost fully maintained after five recycles (with cleaning by ultrasonication between cycles). It was also found that the filtration pressure had little effect on EPS recovery rate and water flux. Subsequently, the removal of heavy-metal ions (HMIs) was achieved via a novel EPS–ENM–DEF process, wherein removal rates of 89.5%, 73.5%, and 74.6% were obtained for Pb^2+^, Cu^2+^, and Cr^6+^, respectively. We, therefore, expect that, on the basis of our results and on the enhanced technology reported herein, further optimization will lead to the preparation of novel and excellent ENMs, e.g., different nanofiber materials and modified techniques, for the highly efficient concentration and recovery of EPSs extracted from excess sludge via CER. This system should also permit the combined removal of HIMs, which will be the focus of our future research in this area.

## Figures and Tables

**Table 1:** Typical parameters for previously reported ENMs fabricated using PVDF over the last 5 years and the PVDF ENMs fabricated in this study *. *d*: pore size; *D*: fiber diameter; *ω*: porosity; *T*: thickness; *θ*: water contact angle; *F*_t_: tensile strength; *ε*: nominal tensile strain at break.

Membrane Fabrication			*d* [μm]	*D* [μm]	*ω* [%]	*T* [μm]	*θ* [°]	*F*_t_ [MPa]	*ε* [%]	Ref.
PVDF [wt.%]	Solvent [*v*/*v*]	Modification [wt.%]								
15	DMF/acetone: 9:1	None	1.82	0.128	68	103	90	1.86	55.2	[47]
15	DMAc/acetone: 4:1	*w*/*h*: 1:1	30	-	36	-	137	0.19	4.8	[32]
		2:1	45		40		134	0.13	3.4	
		3:1	90		44		131	0.08	2.4	
15	DMF/acetone: 3:1	GO: 0	0.8	0.167	-	100	140	6.40	49.3	[48]
		0.5	0.99	0.259			131	5.20	27	
		1	1.13	0.311			119	4.80	12.6	
		3	1.21	0.355			110	4.40	9.5	
15	DMF	SiO_2_NPs: 0	22.00	0.400	-	-	<135	-	-	[49]
		4	22.00	0.600			135			
15	DMAc	None	1.33	-	84	121	92	2.24	13.0	[50]
		f-SiO_2_NPs: 1	1.24		80	116	152	2.13	26.0	
		AgNPs/f-MWCNTs: 2:1	0.64		82	135	56	2.38	30.0	
13.5	DMF/acetone: 6:4	SiO_2_NPs: 0	0.37	0.265	84	107	132	1.83	-	[41]
		2	0.32	0.206	81	110	143	2.16		
		4	0.28	0.209	78	97	149	2.56		
		6	0.27	0.209	77	98	155	1.55		
12	DMF/acetone: 6:4	None	1.30	0.415	-	91	129	-	-	[27]
	DMSO/acetone: 6:4		2.40	0.625		87	130			
	Anhydrous/acetone: 6:4		3.58	0.521		82	116			
12	DMF	PAN: 0	-	0.550	-	-	133	7.125	13.5	[51]
10.8		1.2		0.600			131	9.95	13.0	
6		6		0.550			118	7.40	7.8	
8	NMP	DTPA/MWCNT/TiO_2_: 0	0.006	0.120	-	114	62	0.13	1.8	[31]
		0.5	-	0.116		117	47	0.26	3.5	
		2		0.102		118	38	0.29	5.7	
		6	0.007	0.096		118	27	0.21	6.1	
8	DMF/acetone: 6:4	GO: 0	0.3	0.176	83–85	50	137	-	-	[52]
		5	0.19	0.131			89			
14	DMAc/acetone: 4:1	None	1.47	0.504	52.46	125	109.5	1.54	15.30	This study
18			7.66	0.865	71.53	186	119.1	2.22	20.16	
22			19.36	1.772	73.24	240	129.3	2.58	36.78	

* DMF: dimethylformamide; NMP: *N*-methylpyrrolidone; DMSO: dimethyl sulfoxide; DMAc: dimethylacetamide; NPs: nanoparticles; DTPA/MWCNT/TiO_2_: diethylenetriaminepentaacetic acid (DTPA)-functionalized multiwalled carbon nanotube (MWCNT)/TiO_2_; *w*/*h*: length–width ratio for 3D printing pore geometry; GO: graphene oxide; f-SiO_2_NPs: silanized silica nanoparticles; AgNPs/f-MWCNTs: silver nanoparticles and carboxylated multiwalled carbon nanotubes; PAN: polyacrylonitrile.

## Data Availability

Not applicable.

## Supplementary Materials

The following supporting information can be downloaded at: https://www.mdpi.com/article/10.3390/membranes13010074/s1. Figure S1. Average EPS recovery rates obtained via the UV method (*η*_p-av_) compared with those obtained via the weighing method (*η*_e-av_); Figure S2. Typical images of the water contact angles of the ENMs; Figure S3. Tensile stress–strain curves of the fabricated ENMs; Figure S4. Typical size distributions of the colloids present in the EPS solutions; Figure S5. HIM removal efficiencies during the EPS–ENM–DEF process on ENM-14.
