# Supplementary material for: Recovery of Extracellular Polymeric Substances from Excess Sludge Using High-Flux Electrospun Nanofiber Membranes
Source: Membranes (Basel). 2023 Jan 7;13(1):74. doi: 10.3390/membranes13010074 (PMC9862183; doi:10.3390/membranes13010074)
Supplement: Supplementary file 1 [file membranes-13-00074-s001.zip › membranes-2136376-supplementary.pdf]

Supplementary Material: Recovery of Extracellular Polymeric Substances from Excess Sludge Using High-Flux Electrospun Nanofiber Membranes

Da-Qi Cao [1,*], Xiao-Dan Liu [1,†], Jia-Lin Han [1,†], Wen-Yu Zhang [2], Xiao-Di Hao [1], Eiji Iritani [3] and Nobuyuki Katagiri [4]

[1] Sino-Dutch R&D Centre for Future Wastewater Treatment Technologies/Key Laboratory of Urban Stormwater System and Water Environment, Beijing University of Civil Engineering and Architecture, Beijing 100044, China
[2] Institute of Soil Environment and Pollution Remediation, Beijing Municipal Research Institute of Environmental Protection, Beijing 100037, China
[3] Department of Chemical Engineering, Nagoya University, Furo-cho, Chikusa-ku, Nagoya 464-8603, Japan
[4] Department of Environmental Technology, Meijo University, 1-501 Shiogamaguchi, Tempaku-ku, Nagoya 468-8502, Japan
* Correspondence: caodaqi18@163.com or caodaqi@bucea.edu.cn; Tel.: +86-10-6832-2123
† These authors contributed equally to this work.

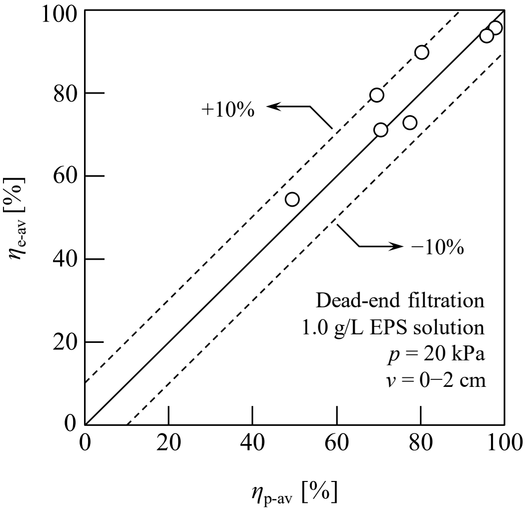

Figure S1. Average EPS recovery rates obtained via the UV method ($\eta_{p\text{-}av}$) compared with those obtained via the weighing method ($\eta_{e\text{-}av}$), wherein the evaluated data were based on for the cumulative filtrate volume per unit membrane area, v = 2 cm.

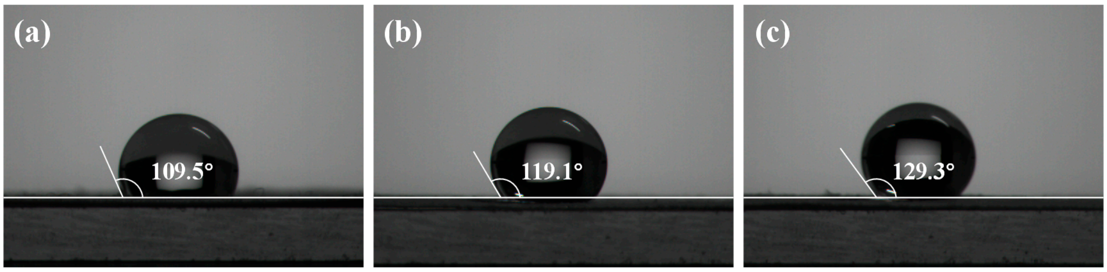

Figure. S2. Typical images of the water contact angles of the ENMs prepared using PVDF at different mass fractions of (a) 14 wt%, (b) 18 wt%, and (c) 22 wt%.

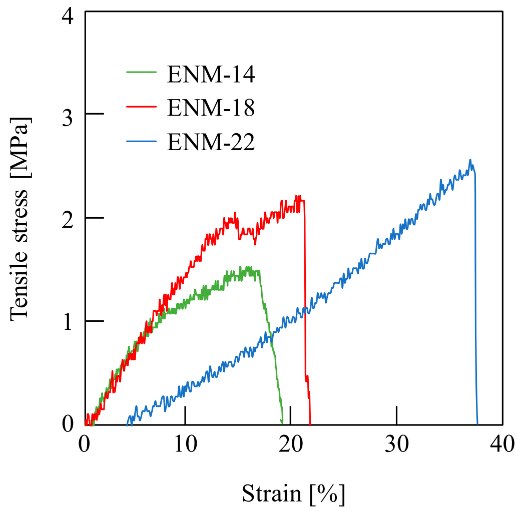

Figure S3. Tensile stress–strain curves of the fabricated ENMs.

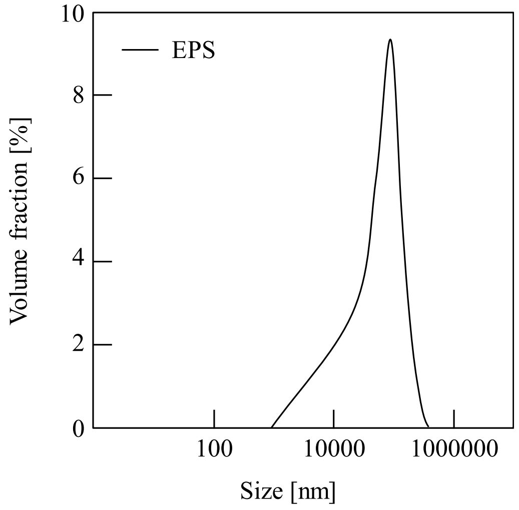

Figure S4. Typical size distributions of the colloids present in the EPS solutions.

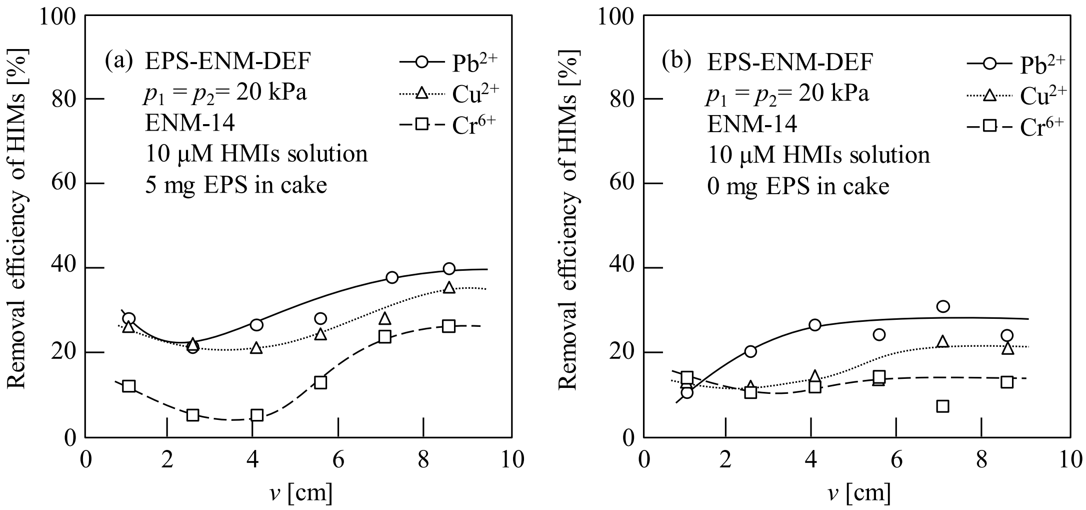

Figure S5. HIM removal efficiencies during the EPS-ENM-DEF process on ENM-14 for (a) 5 mg and (b) 0 mg EPS filter cakes. 1st stage: Concentration and recovery filtration of 0.1 and 0 g/L EPS solutions (50 mL) at p_1 = 20 kPa to give EPS filter cakes with masses of 5 and 0 mg, respectively. 2nd stage: Filtration of 10 μM solutions (180 mL) of the desired HIM (Pb^{2+}, Cu^{2+}, or Cr^{6+}, pH 6.2–6.7) at p_2 = 20 kPa. v is the cumulative filtrate volume per unit membrane area.
